# In Silico Prediction of Potential pTLR7/pSTING Dual-Targeting Ligands via Virtual Screening and Molecular Dynamics Simulation

**DOI:** 10.3390/ijms27010338

**Published:** 2025-12-28

**Authors:** Chang Liu, Zhe Qin, Lixia Bai, Xiao Xu, Wenbo Ge, Zhun Li, Jianyong Li

**Affiliations:** Key Laboratory of New Animal Drug of Gansu Province, Key Laboratory of Veterinary Pharmaceutical Development of Ministry of Agriculture, Lanzhou Institute of Husbandry and Pharmaceutical Science of Chinese Academy Agriculture Sciences, Lanzhou 730050, China; liuchang130183@163.com (C.L.);

**Keywords:** TLR7 ligands, STING ligands, dual-targeting drugs, virtual screening, molecular dynamics simulation

## Abstract

Toll-like receptor 7 (TLR7) and Stimulator of Interferon Genes (STING) ligands possess a series of immunomodulatory effects such as anti-infection, anti-tumor, and autoimmune-disease-alleviating effects. In this study, porcine TLR7 (pTLR7) and porcine STING (pSTING) were selected as targets, and molecular docking and virtual screening methods were used for screening of dual-target livestock immunomodulators. Finally, two compounds were screened with molecular docking scores higher than the positive control compounds. They have good binding ability with pTLR7 and pSTING proteins, as well as satisfactory predictive safety and pharmacokinetic properties. Molecular dynamics (MD) simulation results also indicated that the above ligands can form stable complexes with two target proteins. The average binding free energies of compound **2** with pTLR7 and pSTING were −28.65 kcal/mol and −30.12 kcal/mol, respectively, and of compound **7** with pTLR7 and pSTING were −35.93 kcal/mol and −31.70 kcal/mol, respectively, which were comparable to that of positive control ligands. The similarity of target proteins between pigs, humans, and mice, as well as the interactions between ligands and TLR7 and STING in different species, were analyzed. And analysis of predicted structure–activity relationship (SAR) was conducted. Briefly, compound **2** and compound **7** were predicted to form stable complexes with pTLR7 and pSTING, with satisfactory predicted physicochemical properties and pharmacokinetic characteristics, and represented candidates for experimental validation. This study supplies a research basis for the development, design, and structural modification of immune enhancers for animals.

## 1. Introduction

TLR7 (Toll-like receptor 7) and STING (Stimulator of Interferon Genes) are important components of innate immune responses [1,2], playing crucial roles in the defense against pathogen infection [3,4,5], anti-tumor immune responses [6,7], and cellular autophagy [8,9]. TLR7 is mainly involved in the recognition of single-stranded RNA [10], while STING is related to the inflammatory response triggered by cytoplasmic DNA [11]. Certain pathogen infections [12,13,14,15,16] or tumors [17] can negatively regulate the cGAS-STING or NF-κB signaling pathways, suppress innate immune responses, and thus achieve immune escape. On the other hand, abnormal activation of TLR7 and cGAS-STING signaling pathways may trigger inflammatory responses and autoimmune diseases [18,19].

**Figure 1:**
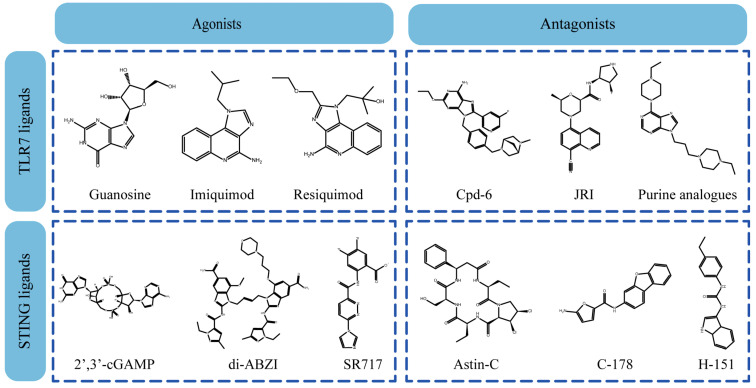
Structure of several representative ligands of TLR7 and STING.

Up to now, a great deal of TLR7 ligands and STING ligands have been synthesized. TLR7 and STING agonists can promote the maturation and activation of immune cells [20] and trigger adaptive immune responses [21,22]. There is tempting potentiality for its immunological enhancement effects [23,24,25,26]. In contrast, TLR7 and STING antagonists are promising to be applied to alleviate inflammation, improve the severity of inflammation, reduce cytokine storms caused by viral infections, and treat autoimmune diseases [18,27]. The structure of TLR7 regulators includes nucleosides and their derivatives [28,29,30], as well as chemically synthesized structures such as imidazo[4,5-c]quinoline [31], pyrido[2,3-d]pyrimidine [32], and quinazoline [33]. Similarly, direct STING ligands also consist of natural ligands and chemically synthesized ligands. For example, cyclic dinucleotides (CDNs) and cyclic peptide Astin C are natural ligands of STING [34,35], and aminobenzimidazoles [36,37], benzothiophenes [38], anthrone [39], quinolone [40], and dibenzofuran [40] belong to chemically synthesized STING ligands. The structures of several TLR7 and STING ligands are shown in Figure 1. The SMILES codes of displayed structures are shown in Table A1.

For many diseases, it is difficult to achieve effective treatment through single-target drugs, while intervening in two or more related targets shows higher therapeutic effects [41]. For example, previous studies showed that the combination of STING agonists and TLR7/8 agonists can cause stronger antigen cross-presentation in tumor immunotherapy, leading to significant tumor regression [42,43], and has a better anti-tumor effect than single-agent therapy. The combined application of STING and TLR7 agonists as vaccine adjuvants is conducive to providing effective immune stimulatory properties, inducing strong and persistent immune responses, better inducing antigen-specific cellular immunity, and producing a balanced Th1/Th2 humoral response [44]. In addition, researchers found that compound **558**, as a single drug, can regulate TLR7/8 and STING, activate inflammasome pathways, and significantly reduce the growth rate of tumor in vivo [45]. Moreover, multi-target drugs have also been extensively studied in the treatment of diseases such as Alzheimer’s disease [46], epilepsy [47], and tumors [48].

However, the activity of TLR7 ligands and STING ligands varies among species. The STING agonist DMXAA is effective against murine STING but has almost no effect on humans [49]. The response of TLR7 from different species to ligands is not equivalent. TLR7/8 agonists, like imiquimod, resiquimod, and gardiquimod, exhibit differential activation effects on TLR7 and/or TLR8 in humans, mice, and pigs [50,51]. In addition, although TLR7 and STING ligands hold great promise for clinical application, the research on livestock and poultry is limited. There is great potential for the development of pTLR7/pSTING dual-targeting drugs.

In an attempt to address this issue, the purpose of this study was to select porcine-derived TLR7 (pTLR7) and STING (pSTING) proteins as targets and preliminarily screen potential dual-targeting ligands through molecular docking and virtual screening methods. This study will lay foundation for the development of immune modulators for livestock and pets in the future, and provides potential solutions for vaccine adjuvants, antiviral and anti-tumor treatments.

## 2. Results and Discussion

### 2.1. Molecular Docking

**Figure 2:**
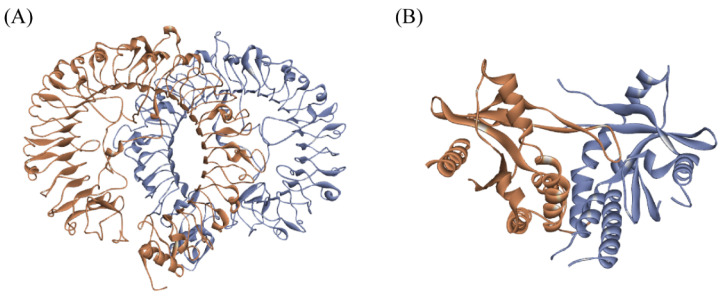
Predicted structure of pTLR7 (**A**) and crystal structure of pSTING (**B**), where orange and purple represent chain A and chain B of protein dimers, respectively.

TLR7 and STING are both transmembrane proteins located on the endoplasmic reticulum. TLR7 binds to ligands and subsequently forms homodimers, transforming into an activated state and regulating downstream signaling pathways [52,53,54]. Similarly, STING also binds specifically to ligands in the form of homodimers, thereby exerting biological functions [55,56]. The structures of the two target proteins, pTLR7 and pSTING, are shown in Figure 2.

Based on the CHARMm force field (within the BIOVIA Discovery Studio (DS) 4.0 software), CDocker is a molecular docking method which can produce high-precision docking results. As described in the literature [57,58], the crystal structures were re-docked before formal docking and the RMSD values were all less than 2 Å (Table A2), indicating the effectiveness of this method in reproducing known binding patterns.

The reported ligands gardiquimod (compound **17**) and SR717 (compound **18**) are positive control compounds for the two targets, pTLR7 and pSTING, respectively. Molecules in Drug-like natural compound library were filtered on the basis of −CDocker energy. Compounds with scores higher than positive controls were retained. In other words, compounds with −CDocker energy higher than gardiquimod (17.64) for pTLR7 and higher than SR717 (34.92) for pSTING were reserved. At this stage, 16 compounds with higher docking scores were screened. The docking scores of the top 16 compounds are shown in Table 1.

### 2.2. ADME and Toxicity Analysis

#### 2.2.1. Physicochemical and ADME Properties Analysis

SwissADME platform was used to evaluate several key ADME features of potential hit compounds [59].

The physicochemical and drug-like properties of the top 16 compounds are presented in Table 2, which includes the molecular weight, number of hydrogen bond donors (HBD), number of hydrogen bond acceptors (HBA), number of rotatable bonds (RB), topological polar surface area (TPSA), bioavailability score, and synthetic accessibility. Among the above compounds, only two compounds (compound **4** and compound **11**) do not follow Lipinski’s rule of five, with two violations, respectively. Other molecules have no violation of Lipinski’s rule of five, indicating that they may be more suitable for oral administration. Lipinski’s rule of five is not a strict standard for natural compounds [60]. Consequently, it is only used as a reference. The bioavailability score is expressed as the probability that a compound has >10% bioavailability or measurable Caco-2 permeability in rats [61]. Four compounds (compound **1**, **3**, **5** and **16**) have the highest bioavailability score, with the value of 0.85. Yet, the bioavailability scores of the two compounds (compound **4** and **11**) are the lowest, with the value of 0.17. The TPSA value less than 140 Å^2^ indicates that compound has high permeability and bioavailability, which corresponds to the ability of the drug to diffuse into cells [62]. The synthetic accessibility score ranges from 1 to 10, which implies the difficulty of synthesis from very easy to very difficult [62,63].

**Figure 3:**
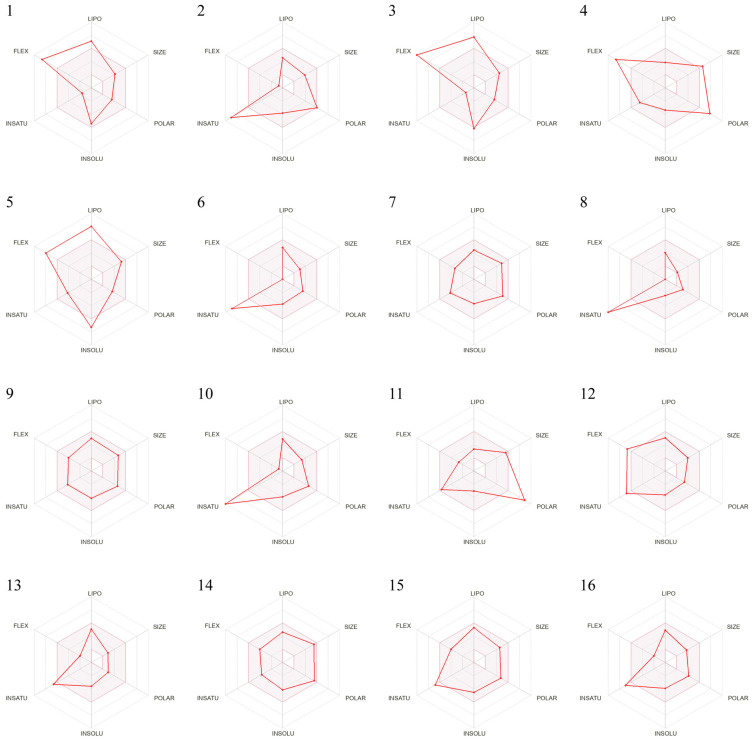
Bioavailability Radar chart of 16 compounds.

The solubility and pharmacokinetic properties of the compounds are shown in Table A3. LogPo/w is the logarithm of the octanol-water partition coefficient, with a best fit range of 0–3. A higher logP value represents higher lipophilicity. It is advantageous for compounds with good lipophilicity to be allocated into the lipid region. There are nine compounds with good lipophilicity, the LogPo/w values of which are within the expected range. In addition, Log S (ESOL) represents water solubility. Two compounds are poorly soluble, and the remaining are moderately soluble or soluble. The Bioavailability Radar chart, generated based on six physicochemical properties, is shown in Figure 3. The pink area is the optimal range of properties.

Except for three compounds (compound **4**, **5** and **11**), all other compounds show high gastrointestinal (GI) absorption. There are a total of five compounds (compound **6**, **8**, **12**, **13** and **16**) with the ability to penetrate the blood–brain barrier (BBB). All compounds are not substrates of P-glycoprotein. The cytochrome CYP-450 microsomal oxygenase system is used to predict the metabolism of molecules. Only compound **11** and compound **16** do not inhibit cytochrome P450 enzymes involved in drug metabolism. This may eliminate the risk of adverse reactions when using these molecules as drugs.

**Figure 4:**
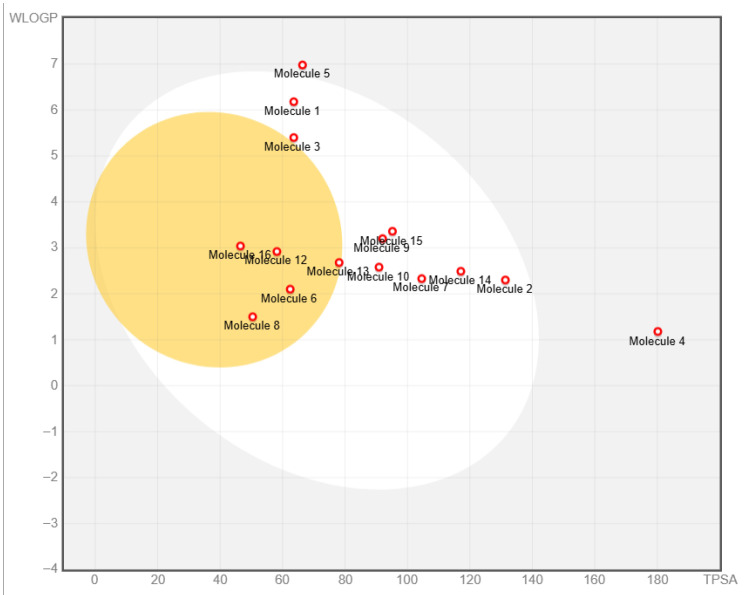
Boiled-egg plot of 16 compounds. Small molecules located within the yolk (the yellow area) indicate their ability to penetrate the blood–brain barrier, while those located within the egg white (grey area) suggest a higher likelihood of being absorbed in the human intestinal tract. The red dots indicate that compounds are not substrates of P-glycoprotein (P-gp).

Meanwhile, boiled-egg plot was also employed to describe the properties of above compounds (Figure 4). Five compounds are located in the yellow area and are expected to penetrate the BBB. And two compounds are in the white region, indicating higher intestinal absorption in humans. All compounds were represented by red dots instead of blue, indicating that they were not substrates of P-glycoprotein [64].

#### 2.2.2. Prediction of Toxicity

Based on the predicted results, 5 out of 16 molecules are non-hepatotoxic, 6 are non-carcinogenic in both female and male rats, 13 are non-mutagenic, and 5 are devoid of developmental toxicity potential (DTP). Apart from that, the rat oral median lethal dose (LD_50_), the rat maximum tolerated dose (LD_0_), and the chronic oral lowest observed adverse effect level (LOAEL) were also predicted. LD_50_ is commonly used in toxicological evaluation. The higher the LD_50_ value, the lower the toxicity of the substance. Chemicals with 500 < LD_50_ ≤ 5000 mg/kg are defined as slightly toxic by the U.S. EPA classification scheme [65,66]. LD_0_ and LOAEL help assess the potential risks of chemical substances and provide a basis for determining safe dosage levels. The relevant parameters of predicted toxicity are listed in Table A4.

In summary, given the physicochemical properties, pharmacokinetic properties and safety, two small molecules, compound **2** and compound **7**, were filtered and analyzed for the interaction with two receptors.

### 2.3. Receptor–Ligand Interaction Analysis

Interactions between receptors and ligands were analyzed. For pTLT7, gardiquimod forms hydrogen bond interaction with THR587 and GLN354* and forms attractive charge and pi-cation interactions with PHE408* and ASP556, respectively. In addition, the formation of pi-pi T-shaped and amide-pi stacked hydrophobic interactions are facilitated by the participation of amino acid residues like LEU558, PHE408* as well as VAL355*. And van der Waals forces dependent on residues such as ASP556, ILE586 and ARG410*. The * symbol indicates that the amino acid residue is located on the B chain of the protein dimer.

**Figure 5:**
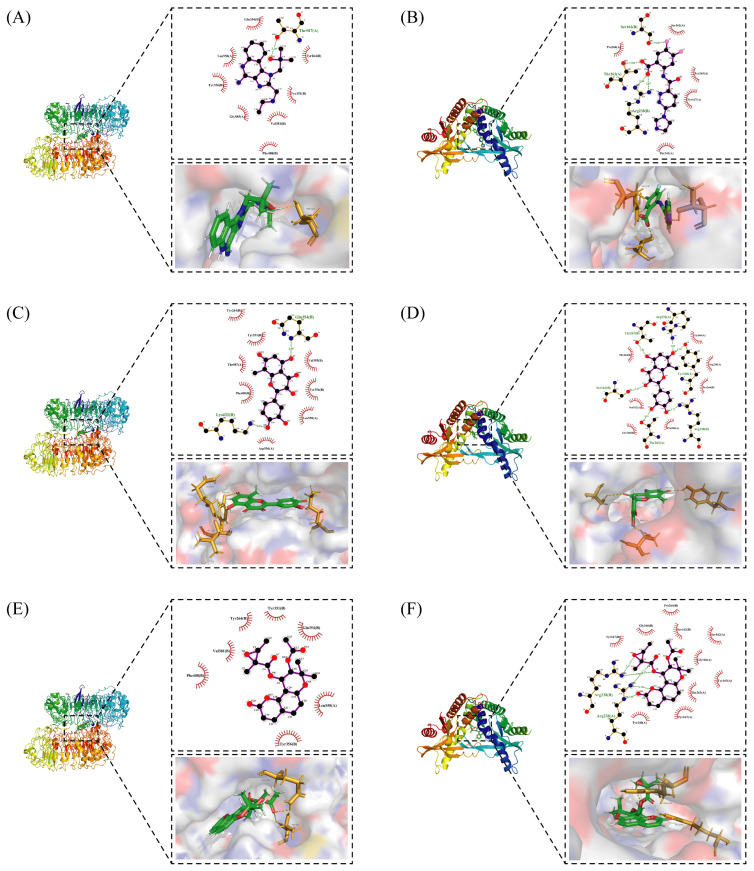
Interactions between receptors and ligands. Interactions between gardiquimod (**A**), compound **2** (**C**), compound **7** (**E**), and pTLR7. (**B**,**D**,**F**) demonstrate the interaction modes between SR717, compound **2**, compound **7,** and pSTING, respectively.

For pSTING and SR717, there is a strong interaction between them. Carboxyl groups and nitrogen atoms of SR717 form hydrogen bonds with the residues SER162*, THR263, ARG238*, GLY166* and THR241, respectively. Meanwhile, ARG238* is also involved in forming attractive charges and pi-cation electrostatic interactions. GLU260, TYR163, PRO264* and other residues are related to van der Waals forces. The pyrazine ring of SR717 contacts with TYR167 through pi-pi stacked hydrophobic interaction. SER162 and GLY166 form halogen with the fluorine atoms of ligand. The receptor–ligand interaction mode diagrams are shown in Figure 5.

#### 2.3.1. Interactions Between Compound **2** and Receptors

Compound **2**, whose IUPAC name is 2-(3,4-dihydroxyphenyl)-3,5,7-trihydroxy-8-methylchromen-4-one, also known as 8-methylquercetin, is a derivative of flavonoids. It has been reported that flavonoids have anti-inflammatory and anti-ulcer activities [67,68]. Flavonoids are effective against many diseases and can be applied for anti-inflammatory, anti-virus, antibacterial [69], and anti-tumor [70] effects, diabetes [71] and so on.

The interactions between compound **2** and pTLR7 and pSTING are shown in Figure 5C,D. Compound **2**, serving as a hydrogen bond acceptor, interacts with TYR351*, GLN354*, VAL355* and THR587 through hydrogen bonds, with LYS432* through salt bridges, and with ALA533, LEU558, and TYR356* through pi-pi stacked and pi-alkyl interactions.

Hydrogen bond interactions are established between compound **2** and residues of pSTING including SER162*, THR263, THR267*, and TYR240. Attractive charge interactions are observed in ARG238, ARG238*, and ARG232. Van der Waals forces are involved with THR263*, GLN266*, and SER162, and a pi-alkyl hydrophobic interaction is formed with PRO264*.

#### 2.3.2. Interactions Between Compound **7** and Receptors

Compound **7**, with IUPAC name of (9-acetyloxy-8,8-dimethyl-2-oxo-9,10-dihydropyrano[2,3-f]chromen-10-yl)2,3-dimethyloxirane-2-carboxylate, belongs to coumarins natural products. Coumarins, which are widely distributed in nature, have been confirmed by clinical research to have pharmacological effects such as anti-inflammatory [72,73], anti-tumor [74], anti-bacterial [75,76] and alleviation of diabetes [77,78].

Compound **7** contacts with GLN354* and THR587 of pTLR7 through hydrogen bonding. Simultaneously, the molecule forms hydrophobic interactions with residues such as TYR356*, LYS432* and LEU558, and is in contact with GLU352*, LEU353* and ASP556 through van der Waals forces.

In its interaction with pSTING, compound **7** forms hydrogen bonds with residues ARG238, ARG238*, and TYR263, pi-cation interactions with ARG238*, hydrophobic interactions with TYR240, TYR163, TYR167*, and TYR167, and is subject to van der Waals forces with amino acid residues such as SER162, PRO264*, THR263*. The mechanism of interactions for compound **7** with pTLR7 and pSTING is illustrated in Figure 5E,F.

### 2.4. MD Simulation Analysis

Molecular dynamics (MD) simulation is a computational chemistry method that simulates the motion and interactions of atoms and molecules over a given period of time. As an open-source software, GROMACS (version 2024.4) has been engaged in a variety of fields owing to its flexibility and powerful capability [79]. GROMACS, based on the principles of mechanics, simulates the motion of any molecule within the system and analyzes the internal interactions and conformational changes in the receptor–ligand complexes. The MD simulation was employed to evaluate the stability of receptor–ligand complexes. The root mean square deviation (RMSD), root mean square fluctuation (RMSF), radius of gyration (Rg), solvent accessible surface area (SASA) and hydrogen bond number (NHB) were analyzed.

#### 2.4.1. RMSD

**Figure 6:**
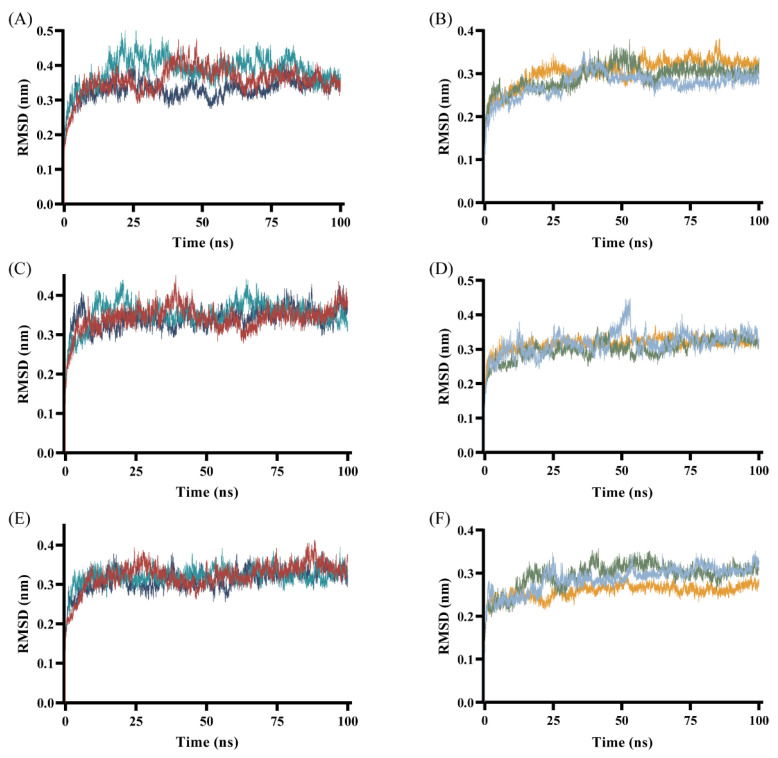
RMSD plot of receptor–ligand complexes. The RMSD curves in the figure reflected the results of three repeated MD simulations. (**A**,**B**) were the RMSD plots of the complexes of pTLR7 and pSTING with their positive control ligands, gardiquimod and SR717, respectively. (**C**,**D**) were the RMSD plots of the complexes of compound **2** with pTLR7 and pSTING, respectively. And (**E**,**F**) were the RMSD plots of the complexes of compound **7** with pTLR7 and pSTING, respectively.

The RMSD curve reflects the positional change of complexes during the simulation process [80]. The smaller the RMSD value, the more stable the structure of complexes. As shown in Figure 6, the complexes had satisfactory stability.

The pTLR7-compound **2** complex gradually stabilizes in the later stage of the simulation, fluctuating within the range of 0.35–0.4 nm (Figure 6C). Similarly, the fluctuation of pTLR7-compound **7** complex is smaller than that of the positive ligand, with more stable RMSD curves (Figure 6E). A stable complex is formed between pSTING and SR717, which gradually stabilized at 40 ns with small fluctuations in RMSD (Figure 6B). The pSTING-compound **2** complex reaches equilibrium at an early stage in the simulation, comparable to that of the positive ligand (Figure 6D). The RMSD of the pSTING-compound **7** complex fluctuates within the range of 0.25–0.35 nm (Figure 6E). Overall, within the given simulation time, the complexes fluctuate within a range of 0.1 nm.

#### 2.4.2. RMSF

The RMSF plot was used to describe the fluctuation of atoms or residues in a molecular system, illustrating the flexibility of atoms or residues [80,81]. As shown in Figure A2, the RMSF of the above complexes fluctuate slightly. The residues 400–600 of pTLR7, and residues 200–250 of pSTING, have greater flexibility.

#### 2.4.3. Radius of Gyration

Rg is often applied to evaluate the tightness and folding stability of the complex through the simulation [57]. Figure A3 demonstrates that the Rg values of complexes formed by pTLR7 and ligands remain stable at around 3.85 nm, while the Rg values of complexes formed by pSTING and ligands remain stable at around 2.20 nm, indicating that the structures of complexes are compact.

#### 2.4.4. Solvent Accessible Surface Area

SASA implies the surface area of the molecular surface that solvent molecules can directly contact [81,82]. According to Figure A4, the surface area of complexes formed by pTLR7 and ligands is around 650–700 nm^2^, while the surface area of complexes formed by pSTING and ligands is in the range of 180–200 nm^2^. Among them, pTLR7 has a relatively large SASA due to its large protein surface area.

#### 2.4.5. H-Bond Numbers

The number of hydrogen bonds formed between target proteins and corresponding ligands are shown in Figure A5. A hydrogen bond interaction is a strong non-covalent interaction, which is beneficial to maintaining the stability of complexes [83]. There are strong hydrogen bonding interactions between pTLR7-gardiquimod complex and pSTING-SR717 complex, with 0–4 and 0–6 hydrogen bonds within the simulation time of 0–100 ns, respectively. With both target proteins, compound 2 forms two or fewer hydrogen bonds. Comparatively, compound **7** has more hydrogen bonds with two targets, ranging from 0 to 4, respectively.

#### 2.4.6. Comparison of Complex Conformations

**Figure 7:**
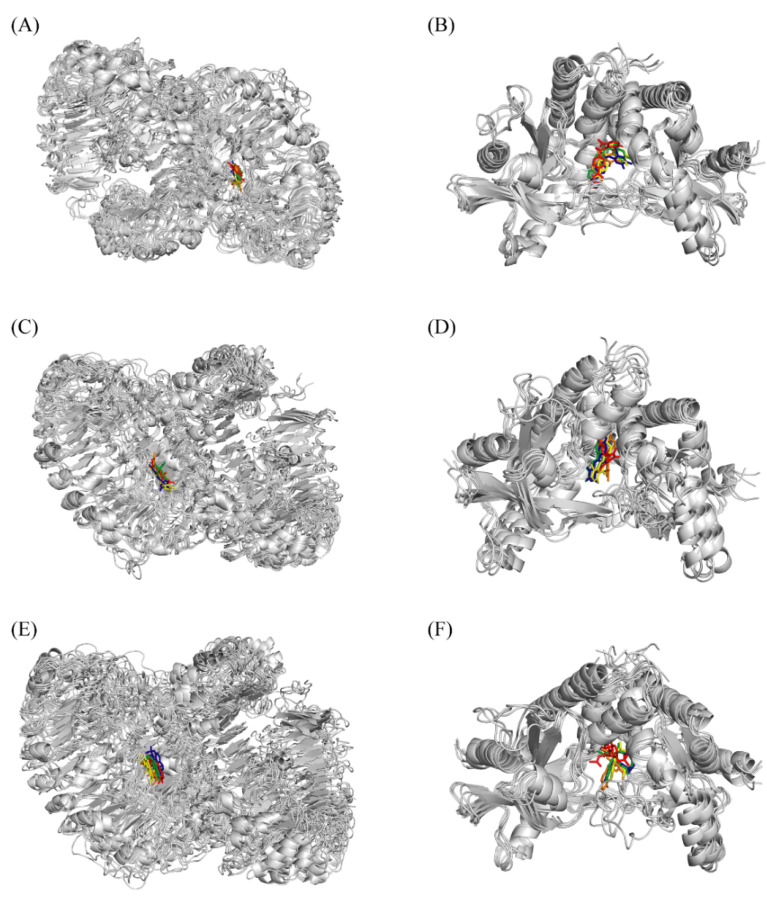
Conformation of complexes at different time points. The conformations of pTLR7-gardiquimod complex, pSTING-SR717 complex, pTLR7-compound **2** complex, pSTING-compound **2** complex, pTLR7-compound **7** complex and pSTING-compound **7** complex were displayed sequentially from (**A**–**F**). The small molecules in red, green, blue, yellow, and orange correspond to the structures of ligands at 0 ns, 25 ns, 50 ns, 75 ns, and 100 ns, respectively.

By comparing the conformations at different times during MD simulation, the binding stability of complexes was investigated. As can be seen in Figure 7, compound **2** and compound **7** remain bound to the active pocket of pTLR7 and pSTING, which is similar to positive control ligands, suggesting a desirable binding stability of the formed complexes. In Figure 7, the small molecules in red, green, blue, yellow, and orange correspond to the structures of ligands at 0 ns, 25 ns, 50 ns, 75 ns, and 100 ns, respectively. In addition, the center-of-mass (CoM) distances between ligands and active sites at 0 ns, 25 ns, 50 ns, 75 ns, and 100 ns were calculated, as shown in Table A5.

#### 2.4.7. Gibbs Energy Landscape

The Gibbs energy landscape was calculated using the g-sham script in Gromacs. RMSD and Rg values were employed for calculation, and subsequently, the landscape map was generated. The landscape map is used to describe the conformation of complex with the minimum energy during dynamic simulation. In Figure A6, the purple and blue region reflect the lower energy value, indicating the more stable structure. On the contrary, the red area represents unstable structures [80,84,85]. The free energy distribution of complex exhibits a clear minimum value, indicating that the system has high thermodynamic stability in this conformation and is kinetically reasonable. Gibbs energy landscapes indicate that the complexes formed between compound **2** and compound **7** and pTLR7 and pSTING have good stability.

### 2.5. MM-GBSA Calculation

MM-GBSA binding free energy was calculated based on three replicas of MD simulations to assess the binding affinity of complexes. Molecular mechanics terms and solvation energy, including Van der Waals, electrostatic, polar resolution, SASA energy, as well as average binding free energy were calculated [86,87,88]. The average binding free energy (Δ*G*_bind_) of protein–ligand complex was calculated according to the following equation:

$${\Delta G}_{bind}={\Delta E}_{vdw}+{\Delta E}_{ele}+{\Delta G}_{GB}+{\Delta E}_{SA}$$

where Δ*E*_vdw_, Δ*E*_ele_, Δ*G*_GB_ and Δ*G*_SA_ stand for van der Waals energy, electrostatic energy, polar and nonpolar components of the desolvation free energy, respectively.

Binding free energy of complexes are illustrated in Table 3. The average binding free energies of the complex formed by compound **2** with pTLR7 and pSTING are −28.65 kcal/mol and −30.12 kcal/mol, respectively. Likewise, the average binding free energies of compound **7** with pTLR7 and pSTING are −35.93 kcal/mol and −31.70 kcal/mol, respectively. The binding free energies of screened ligands are comparable to that of positive control ligands, demonstrating strong interactions to target proteins.

### 2.6. Similarity Analysis and Sequence Alignment

In order to further analyze the homology of porcine targets with humans and mice, as well as the binding characteristics between ligands and receptors from different species, sequence alignment and similarity analysis were conducted.

**Figure 8:**
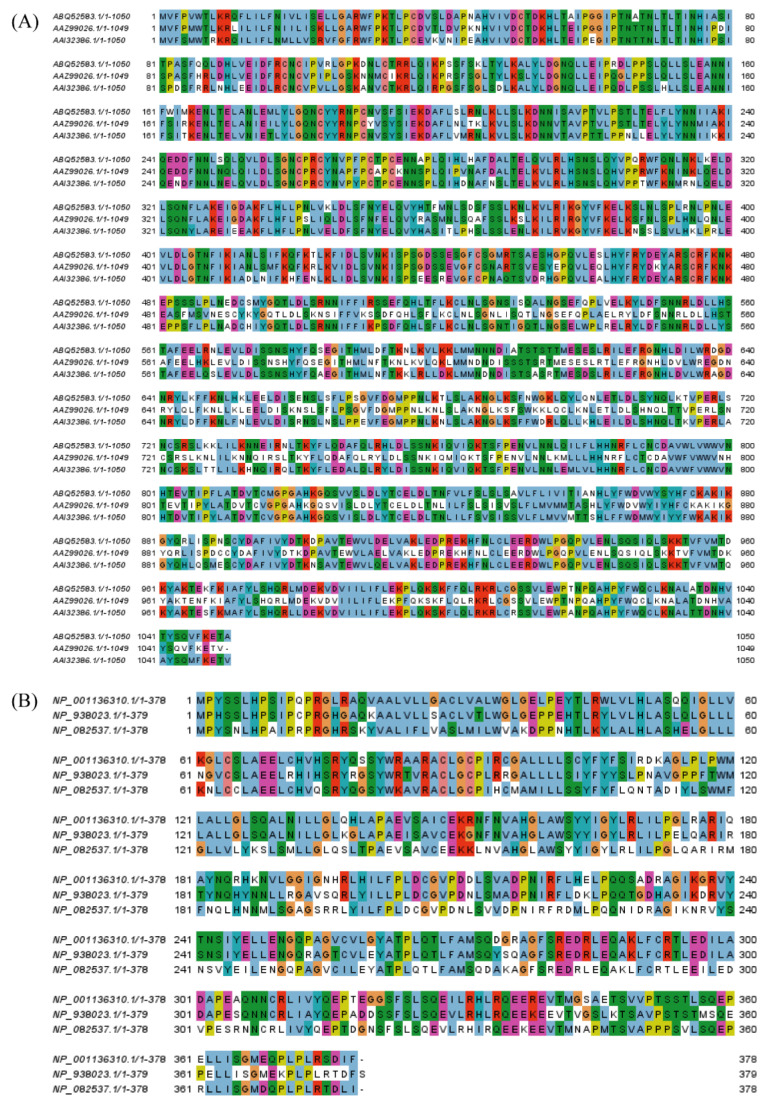
Sequence alignment. (**A**) Sequence alignment of TLR7 between pigs, humans, and mice. (**B**) Sequence alignment of STING between pigs, humans, and mice. Amino acids were colored based on their properties: blue for hydrophobic, red for positive charge, magenta for negative charge, green for polar, pink for cysteines, orange for glycines, yellow for prolines, cyan for aromatic, and white for unconserved residues.

The sequence identity of TLR7 (Accession: ABQ52583.1) and STING (Accession: NP_001136310.1) between pigs, humans, and mice was analyzed using Basic Local Alignment Search Tool (BLAST version 2.17.0), and sequence alignment was performed. The percent identity of TLR7 between pigs, humans, and mice is 84.95% and 78.29%, respectively. The percent identity of STING between pigs, humans, and mice is 76.98% and 69.92%, respectively. The identity and BLAST results are shown in Table A6. And sequence alignment results can be seen in Figure 8.

### 2.7. Interactions Between Ligands and Receptors of Different Species

**Figure 9:**
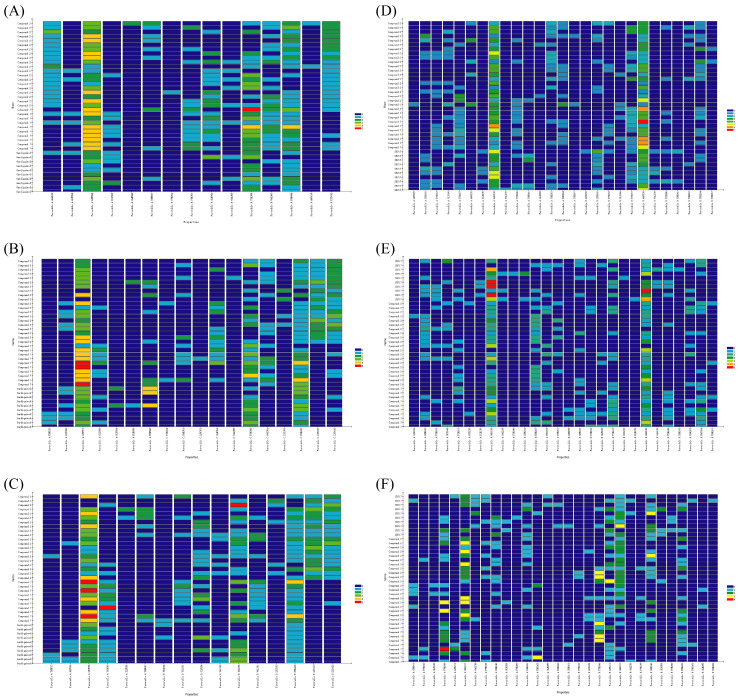
Interaction heatmap. (**A**–**C**) were interaction heatmaps of gardiquimod, compound **2,** and compound **7** with TLR7 in pigs (**A**), humans (**B**), and mice (**C**), respectively. (**D**–**F**) were interaction heatmaps of SR717, compound **2,** and compound **7** with STING in pigs (**A**), humans (**B**), and mice (**C**), respectively.

**Figure A1:**
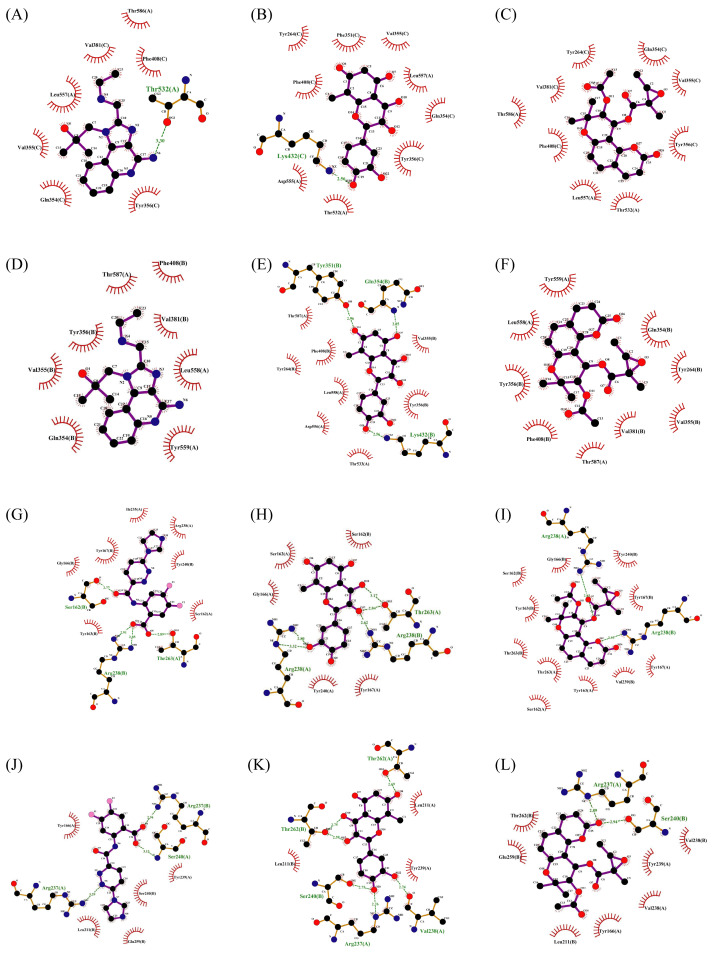
Interactions between ligands and receptors in humans and mice. From (**A**–**C**) were interactions between gardiquimod, compound-2, compound-7, and human TLR7, respectively. From (**D**–**F**) were interactions between gardiquimod, compound-2, compound-7, and murine TLR7, respectively. From (**G**–**I**) were interactions between SR717, compound-2, compound-7, and human STING, respectively. From (**J**–**L**) were interactions between SR717, compound-2, compound-7, and murine STING, respectively.

**Figure A2:**
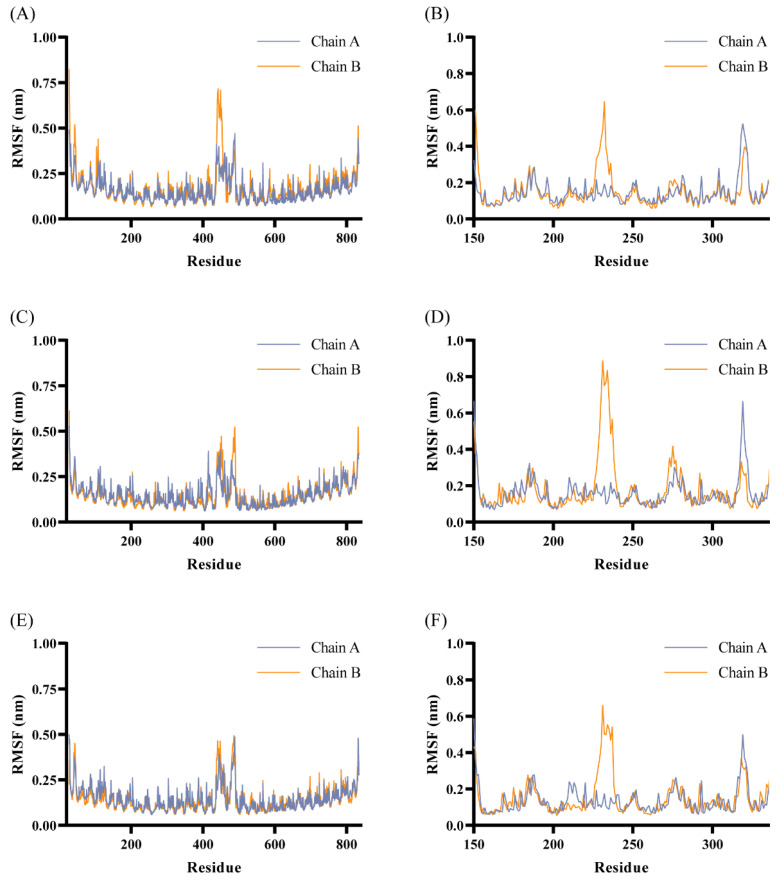
RMSF plot of receptor–ligand complexes. (**A**–**F**) showed the RMSF plot of pTLR7-gardiquimod, pSTING-SR717, pTLR7-compound **2**, pSTING-compound **2**, pTLR7-compound **7**, pSTING-compound **7** complexes in sequence.

**Figure A3:**
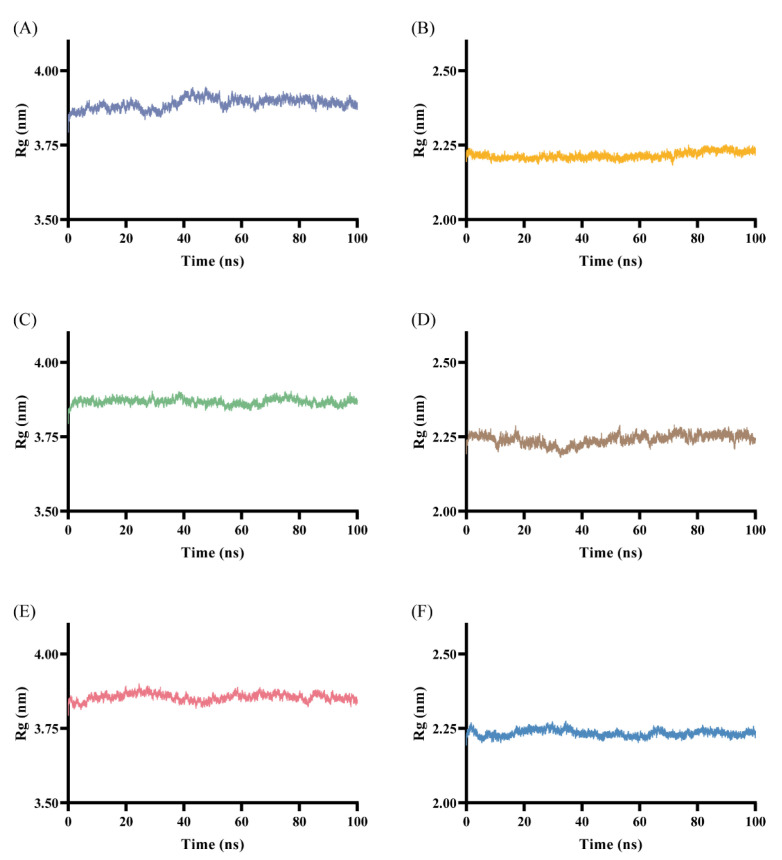
Rg plot of receptor–ligand complexes. (**A**–**F**) showed the Rg plot of pTLR7-gardiquimod, pSTING-SR717, pTLR7-compound **2**, pSTING-compound **2**, pTLR7-compound **7**, pSTING-compound **7** complexes in sequence.

**Figure A4:**
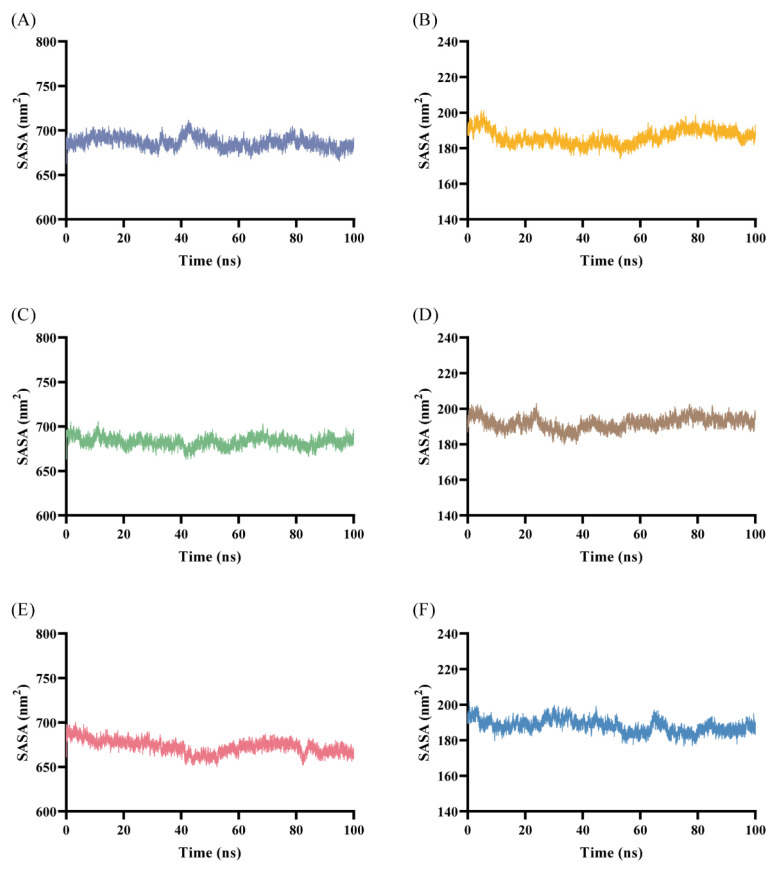
SASA curves of receptor–ligand complexes. (**A**–**F**) showed the SASA plot of pTLR7-gardiquimod, pSTING-SR717, pTLR7-compound **2**, pSTING-compound **2**, pTLR7-compound **7** and pSTING-compound **7** complexes in sequence.

**Figure A5:**
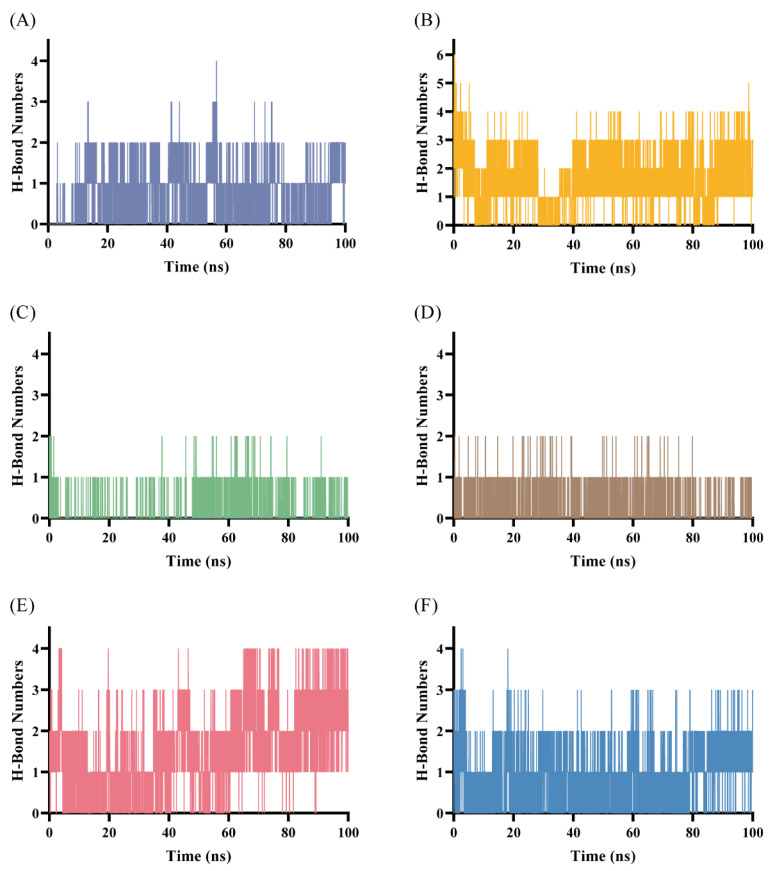
Numbers of H-bond. (**A**–**F**) showed the H-bond numbers of pTLR7-gardiquimod, pSTING-SR717, pTLR7-compound **2**, pSTING-compound **2**, pTLR7-compound **7,** and pSTING-compound **7** complexes in sequence.

**Figure A6:**
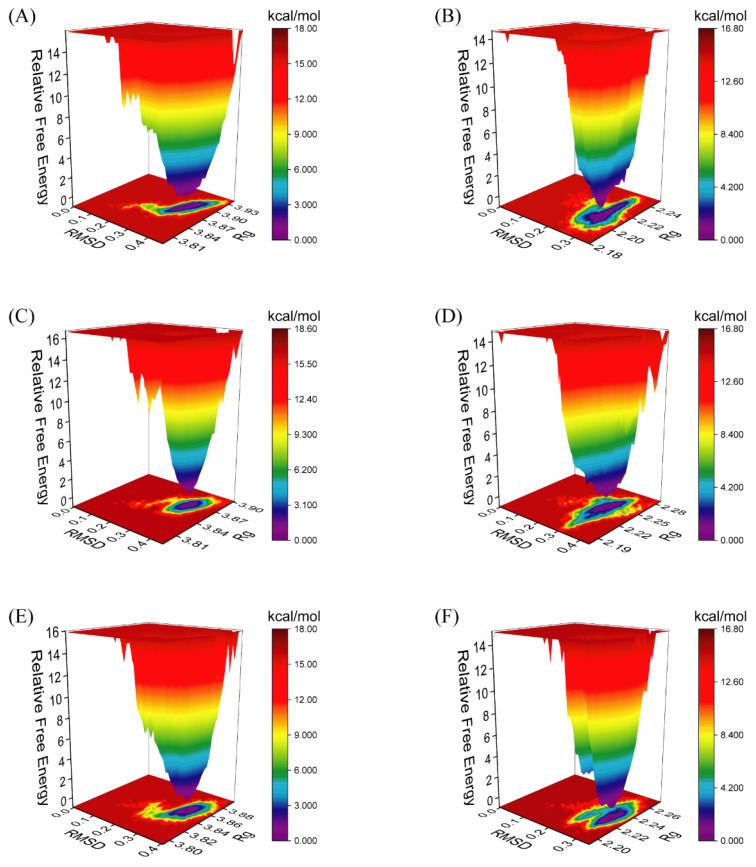
Gibbs energy landscape. From (**A**–**F**) were pTLR7-gardiquimod complex (**A**), pTLR7-compound **2** complex (**B**), pTLR7-compound **7** complex (**C**), pSTING-SR717 complex (**D**), pSTING-compound **2** complex (**E**), and pSTING-compound **7** complex (**F**), respectively.

Furthermore, the binding characteristics between ligands and receptors in humans and mice were analyzed, aiming to explore potential differences in their interactions with these targets across different species. The interaction mode between the receptor in humans and ligands is depicted in Figure A1, and the interaction heatmap can be seen in Figure 9.

For human TLR7, LYS432* and PHE408* amino acid residues interact with gardiquimod through hydrogen bonding or pi-cation, which is a type of electrostatic interaction. THR586, GLN354*, VAL355 and other residues are connected to compound **2** through hydrogen bonding, while LYS432* and LYS410* participate in the formation of salt bridges. Compound **7** forms hydrogen bonds with THR586 and GLN354* residues of human TLR7 and forms hydrophobic interactions with residues such as TYR356* (Figure A1A–C).

For TLR7 in mice, THR533, THR587, and GLN354* form hydrogen bonds with gardiquimod, while residues ASP556, TYR351*, and PHE408* are related to electrostatic interactions. There is a strong interaction between murine TLR7 and compound **2**. It forms hydrogen bonds with residues THR587, TYR351*, GLN354* and VAL355*, salt bridges with LYS410* and LYS432*, and interacts with LYS383* through attractive charge. In addition, THR587, TYR559, and GLN354* residues of murine TLR7 are linked to compound **7** through hydrogen bonding (Figure A1D–F).

As can be seen in Figure 9A–C, in human TLR7, LEU557, and TYR356* exhibit significant involvement in the interaction with ligands, resulting in numerous favorable interactions. In pigs and mice, LEU558 and TYR356* also contribute significantly to binding by forming hydrophobic and charge interactions, as well as hydrogen-bonding interactions. VAL381* interacts more frequently with three ligands, mainly through hydrophobic contacts, while the engagement of this residue is weaker in humans and mice.

For human STING, it forms hydrogen bonds with SR717 through amino acid residues including SER241, THR263, and SER162*, forms halogen bonds through GLY166 and THR263, and forms salt bridge and pi-cation interaction through ARG238 and ARG238*. Similarly, SER162, ARG238, THR263, and THR267 of human STING come into contact with compound **2** through hydrogen bonding, and ARG238* forms an attractive charge with it. Strong hydrogen bonding interactions are also observed between human STING and compound **7** (Figure A1G–I).

In terms of STING in mice, it relies on residues such as SER240 and ARG237 to form hydrogen bonds with SR717, forming salt bridge with GLU259 and ARG237*, and halogen bonds with fluorine through residues including VAL238, TYR260, and VAL238*. Its residues such as ARG237, VAL238, TYR162*, and SER240* interact with compound **2** through hydrogen bonding, ARG237 and ARG237* residues are associated with the formation of attractive charge. Hydrogen bonds are observed between residues ARG237, THR262, and SER240* and compound **7** (Figure A1J–L).

For STING, residues ARG238 and ARG238* in humans and pigs, along with ARG237 and ARG237* in mice, engage in strong interactions with the above ligands, involving hydrogen bonds, electrostatic and hydrophobic forces. Furthermore, these residues can also form halogen bonds with SR717. The TYR166 residue in mice has strong interactions with SR717, compound **2**, and compound **7**, but its involvement is relatively weak in both humans and pigs (Figure 9D–F).

Overall, the key amino acids involved in interactions between receptors in different species and ligands are similar or consistent, but there are still a few differences. The differences in key amino acids between species are of great significance for the design of species-specific drugs.

### 2.8. Prediction of Structure–Activity Relationship (SAR) of Compound **2** and Compound **7**

Through methods such as substituent changes and scaffold hopping, ten and nine derivatives were designed based on compound **2** and compound **7**, respectively. The structures of derivatives and their docking scores with two receptor proteins are listed in Table 4.

**Figure 10:**
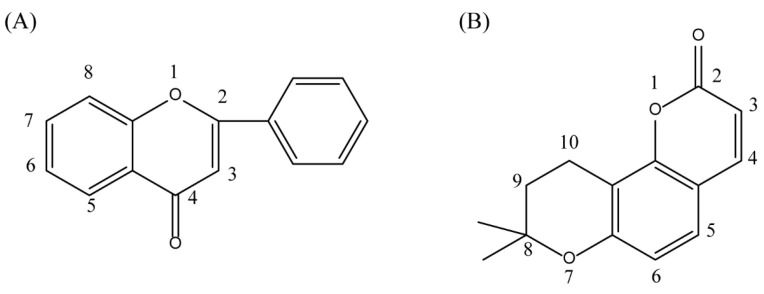
Core structure of compound **2** (**A**) and compound **7** (**B**).

The parent nuclear structure of compound **2** is 2-phenylchromenone (Figure 10A). Derivatives **2-1** to **2-10** investigate the effects of changes in the skeleton and side chain substituents on receptor–ligand interactions.

**Figure 11:**
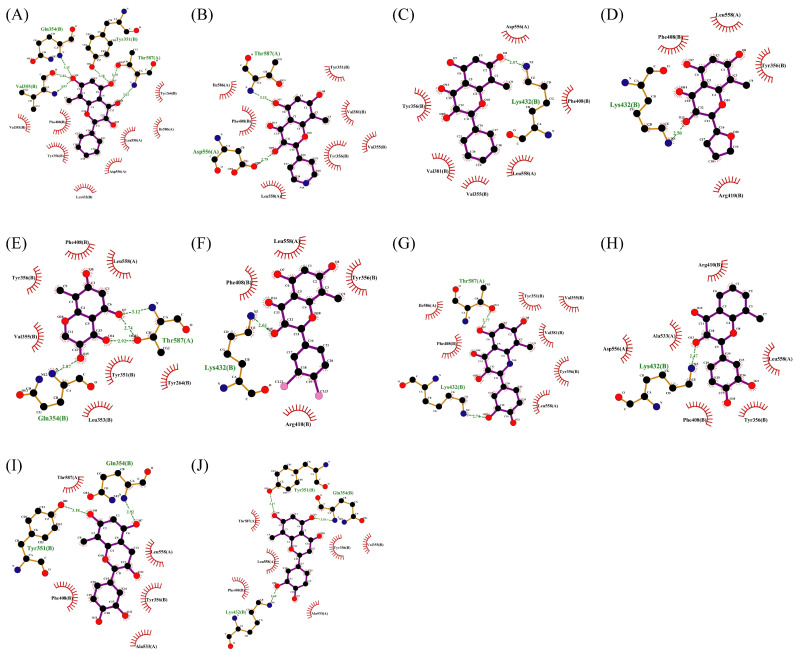
Interactions between 10 derivatives of compound **2** (**2-1** to **2-10**) with pTLR7 were shown in sequence from (**A**–**J**).

**Figure 12:**
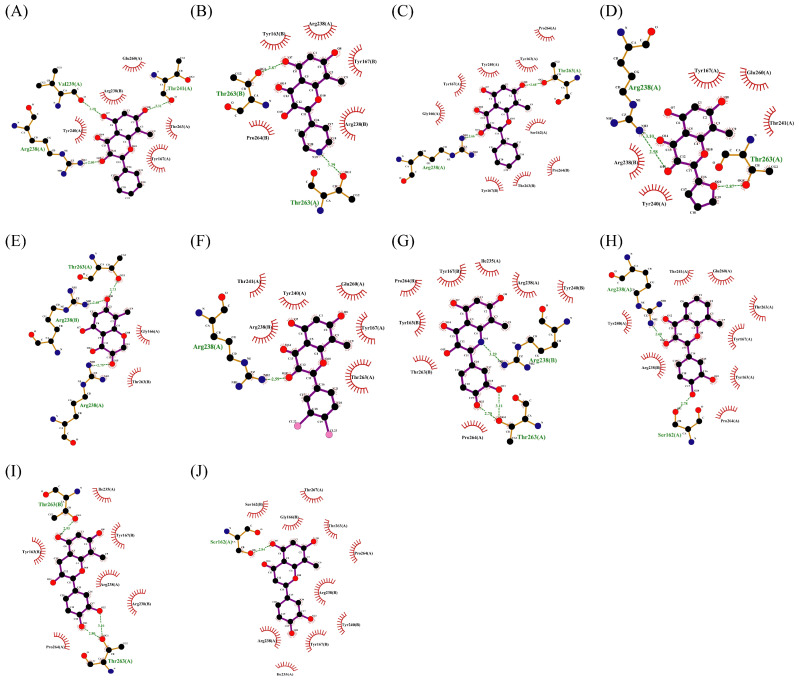
Interactions between 10 derivatives of compound **2** (**2-1** to **2-10**) with pSTING were shown in sequence from (**A**–**J**).

The changes in the phenyl substituent at the 2-position (structure **2-2** to **2-5**) and the removal of carbonyl group at the 4-position (**2-9**) do not show positive effects on improving the docking scores and interactions. It is worth noting that, compared to compound **2**, the docking score with pSTING did not decrease after removing the phenyl substituent at the 2-position (**2-5**), which may indicate that the structure of flavonoids could be altered as pSTING ligands. Derivatives **2-1** and **2-6** are modified based on the hydroxyl substituent on the phenyl group, but the docking scores decreased. This probably means that the electron-donating group at that position is more conducive to binding with receptors. Converting the alkoxyl group to imino group (**2-7**), the docking scores of the derivative with pTLR7 and pSTING remain almost unchanged. The removal of hydroxyl groups at the 6-position and 8-position (**2-8**) result in varying degrees of decrease in docking scores. The exclusion of hydroxyl group at the 3-position (**2-10**) has little effect on the interaction with pTLR7. But it has a more significant impact on the interaction with pSTING. The interactions between 10 derivatives of compound **2** and pTLR7 and pSTING are depicted in Figure 11 and Figure 12.

**Figure 13:**
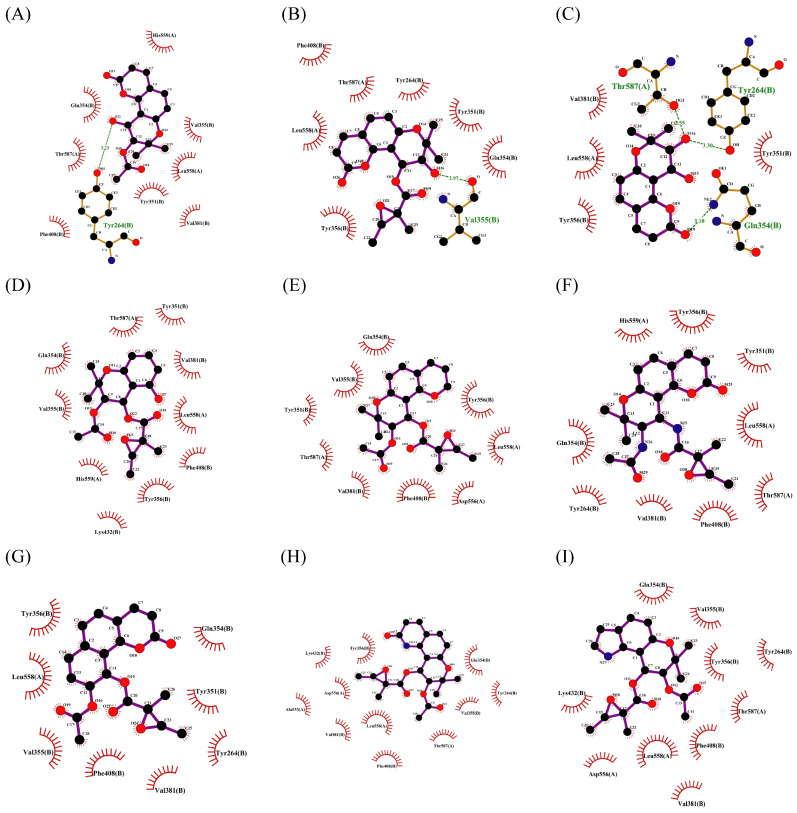
Interactions between 9 derivatives of compound **7** (**7-1** to **7-9**) with pTLR7 were shown in sequence from (**A**–**I**).

**Figure 14:**
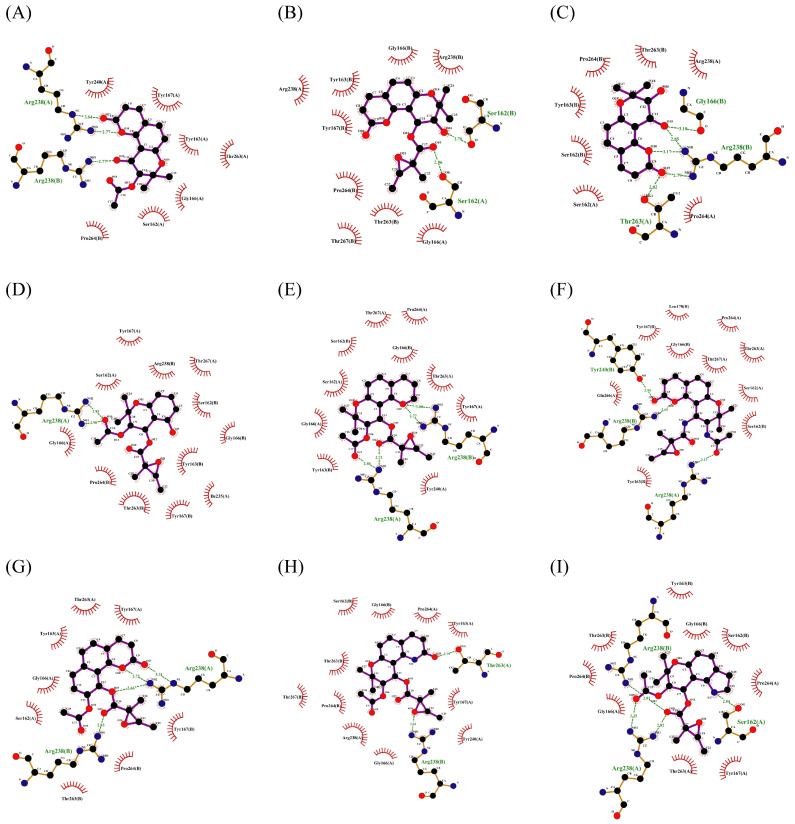
Interactions between 9 derivatives of compound **7** (**7-1** to **7-9**) with pSTING were shown in sequence from (**A**–**I**).

Dihydroseselin could be considered as the main structure of compound **7** (Figure 10B). As depicted in Table 4, derivatives **7-1** to **7-3**, as well as **7-6**, are used to investigate the influence of ester groups at the 9- and 10-positions on receptor–ligand interactions. The exclusion and alteration of ester groups result in a decrease in docking scores or weakening of interactions. However, the modification from ester group to amide group (**7-6**) causes little change in the interaction with pTLR7. In addition, derivatives **7-4** to **7-5**, as well as **7-7** to **7-9**, are used to investigate the role of heterocycles in their interactions with receptors. It is found that the docking scores and interactions of **7-8** and **7-9** with pTLR7 are comparable to compound **7**. In contrast, after changing the heterocyclic ring of the main scaffold, the derivatives still show strong interactions with pSTING, but the docking scores decreass to varying degrees. It is speculated that this phenomenon might be related to unfavorable steric hindrance and the loss of hydrophobic interactions. Fused heterocycles may play a significant role in the interaction between ligands and pSTING. The interactions between the nine derivatives of compound **7** and pTLR7 and pSTING are shown in Figure 13 and Figure 14.

## 3. Limitations and Future Research Directions

At present, pharmacological research has been upgraded from mechanism description to target intervention, achieving a closed-loop study of “mechanism–target–drug”. Although multi-target drugs can intervene in multiple pathological stages of diseases comprehensively, they face challenges in development process. For example, balancing the activity between different targets, the complexity of structural optimization, and the costs far exceeding those of single target drugs are all issues that need to be considered. In addition, the activity detection of TLR7 and STING agonists/antagonists is usually based on methods such as reporter gene analysis or molecular interaction analysis. However, due to the large workload and high cost of constructing reporter cells and obtaining two target proteins, the affinity between ligands and target proteins has not been validated at present. In the later stage, the interaction between targets and small molecules, as well as the immunomodulatory effect and mechanism of above compounds in vitro and in vivo will be further explored.

## 4. Materials and Methods

### 4.1. Information Collection and Data Retrieval

The three-dimensional structure of pTLR7 and murine TLR7 were predicted by SWISS-MODEL online platform (https://swissmodel.expasy.org/) (accessed on 6 December 2024) in our previous work (unpublished paper) and were considered to have reliable quality. The crystal structure of monkey TLR7 (PDB ID: 5GMF, resolution 2.50 Å) was selected as modeling template [53]. The structure of pSTING was downloaded from the RCSB PDB protein databank (https://www.rcsb.org) (accessed on 6 December 2024) (PDB ID: 6IYF, resolution 1.76 Å) [89]. The crystal structure of monkey TLR7 (PDB ID: 5GMF) was used as a substitute for human TLR7 and was further analyzed [53]. The structures of human STING (PDB ID: 7SHP) [90] and murine STING (PDB ID: 4KBY) [91] were all downloaded from PDB website. The compound information of the Drug-like natural compound library (522 compounds) was provided by TargetMol, Boston, MA, USA. The structural information of gardiquimod and SR717 was downloaded from the PubChem (https://pubchem.ncbi.nlm.nih.gov) (accessed on 9 September 2024).

### 4.2. Sequence Research and Alignment

The information of amino acid sequences was downloaded from NCBI (https://www.ncbi.nlm.nih.gov/) (accessed on 18 September 2025). Sequence identity was analyzed in BLAST (https://blast.ncbi.nlm.nih.gov/Blast.cgi) (accessed on 18 December 2025). And sequence alignment was performed in Jalview software (version 2.11.2.0).

### 4.3. Preparation of Proteins and Definition of Site Sphere

According to the previously reported method, the receptors and ligands were processed in DS software, separately [92]. The proteins, pTLR7 and pSTING, were defined as receptors and processed by Prepare protein module, respectively. The heteroatoms and water molecules were removed. H atoms were added and the missing amino acid residues were supplemented. And the pH for protonation was 7.4.

Generation of the docking pocket is a crucial step, as it specifies the protein region where ligands will dock and provides important impact on the calculation of docking scores. In this study, based on previous research, gardiquimod and SR717 were selected as the positive control ligands for pTLR7 and pSTING, respectively. The position of the spherical cavity that accommodates known ligands was defined as the docking site sphere. The central coordinates of the site sphere of pTLR7 receptor were X = 24.463553, Y = −6.949464, Z = 32.1999381, and for pSTING were X = −3.791994, Y = 0.824487, Z = −30.206297, both with a radius of 20 Å.

### 4.4. Preparation of Ligands

Ligands were processed using the Prepare Ligand module to remove duplicate conformations, enumerate isomers and tautomers, and generate 3D conformations, and H atoms were added. The atomic charges of small molecules were calculated based on CHARMm force field. In addition, pH-based ionization method was used with a range of 7.0 to 7.4. With 522 molecules inputted, a total of 29,580 ligands were created and 584 ligands were rejected.

### 4.5. Molecular Docking

Molecular docking was performed through CDocker module of DS 4.0 software. The docking result was represented as −CDocker Energy, which includes receptor–ligand interaction energy and ligand strain energy. The receptor was held rigid while the ligands were allowed to flex. Corresponding proteins were defined as receptors, respectively, and the processed compounds were inputted as ligands. The site sphere set above served as docking pocket, respectively. Post cluster radius of Top Hits was set to 0.5 to ensure maximum diversity of docking conformations. Random conformation was set to 10, indicating each ligand will generate 10 poses after molecular docking. The grid spacing value was set to the default of 0.5 Å [93]. Other parameters were set as default. LigPlot+ (version 2.3.1) and Pymol (version 2.4.0) software were employed for receptor–ligand interaction analysis.

### 4.6. Prediction of ADME Property and Toxicity

The physicochemical properties of compounds were evaluated by SwissADME (http://www.swissadme.ch/index.php) (accessed on 27 February 2025), including the lipophilicity, water solubility, pharmacokinetics proficiency, drug-likeness, and pharmaceutical chemistry [62,94]. The lipophilicity and oral bioavailability radar charts were generated according to the predicted results.

Potential toxic effects of compounds were predicted by the Toxicity module in DS, including liver toxicity, rat oral LD_50_, rat maximum tolerated dose, rodent carcinogenicity, Ames mutagenicity, rat chronic oral LOAEL, as well as DTP.

### 4.7. Molecular Dynamics Simulation and MM-GBSA Calculation

GROMACS was used for MD simulations and further analysis. The procedure, which generally consists of four parts—preparation, setup, simulation, and analysis—was moderately adjusted according to the tutorial and the literature [79,80,95].

The conformation with the highest score in molecular docking was selected for MD simulation, and Amber14sb and Gaff2 force field were used to generate protein and ligand topology files, respectively. The atomic charges of small molecules were calculated using the ACPYPE tool. ACPYPE employed the AM1-BCC method to automatically compute partial atomic charges, and subsequently complete topology files were generated. The TIP4P water model was applied for solvation of the complex system, and a water box with a periodic boundary of 1.2 nm was established. The Particle Mesh Ewald (PME) method was used to treat long-range electrostatic interactions. A Verlet cutoff scheme was applied for neighbor searching. Bonds involving H atoms were holonomic constrained using the LINCS algorithm. Chloride and sodium ions with final concentration of 0.15 mol/L were added to neutralize the system, as well as to simulate the in vivo environment. Steepest descent method was adapted to minimize energy in 50,000 steps, with the intention of eliminating poor atomic contacts. Canonical ensembles (NVT) equilibration simulation was performed for 100 ps, stabilizing the system from initial state to 310 K. -DPOSRES was used to apply position restraint to the protein and ligand. Isothermal isobaric ensembles (NPT) equilibration simulation was performed for 100 ps to achieve thermodynamic equilibrium of the system at 310 K and 1 bar. The final volume and density of the system approached physiological conditions. The key parameters of NPT equilibration simulation were consistent with NVT to ensure the coherence of simulation parameters and computational consistency. A 100 ns MD simulation was conducted with a time step of 2 fs under conditions of 310 K and 1 bar, and the structural coordinates were saved every 10 ps.

Molecular Mechanics/Generalized Born Surface Area (MM/GBSA) analysis can be engaged in to estimate the binding free energy of receptor–ligand complexes, which is crucial for understanding molecular recognition processes and drug design [96]. GROMACS software and the gmx_mmpbsa tool was applied to calculate MM-GBSA of protein–ligand complex.

Finally, the results were analyzed through RMSD, RMSF, Rg, SASA, and NHB, as well as the Gibbs energy landscape to elucidate the dynamic behavior of proteins.

## 5. Conclusions

This study preliminarily screened two drug-like natural compounds, belonging to flavonoids and coumarins, as predictive immunomodulators through virtual screening.

These two compounds have better molecular docking scores than positive control ligands and exhibit good physicochemical properties and pharmacokinetic characteristics. Both the interaction analysis and MD simulation indicate that strong interactions were formed between receptors and ligands, which serve as main contributors to the stabilization of complexes. The calculated average binding free energies are higher than that of positive reference molecules, which are −30.26 kcal/mol and −34.06 kcal/mol against pTLR7 and pSTING for compound **2**, and −35.09 kcal/mol and −30.70 kcal/mol for compound **7**.

Although biological evaluation has not yet been conducted due to the unavailability of the compounds, the computational evidence of this work provides a strong theoretical basis for the high potential of these two natural derivatives as immunomodulators. This study provides a research foundation for the development of the two natural product derivatives as immunomodulators, as well as their application in the prevention and treatment of livestock and poultry diseases. In future work, the biological activity, SAR analysis, and structural modification, as well as their immunomodulatory properties, will be further explored.

## Figures and Tables

**Table 1:** Chemical structure and the −CDocker energy of top 16 compounds.

No.	Structure	−CDocker Energy (kcal/mol)		No.	Structure	−CDocker Energy (kcal/mol)	
		pTLR7	pSTING			pTLR7	pSTING
**1**	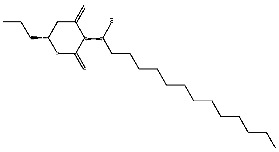	55.30	75.94	**10**	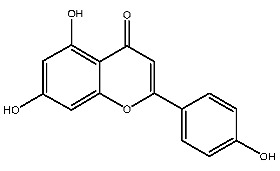	44.38	43.51
**2**	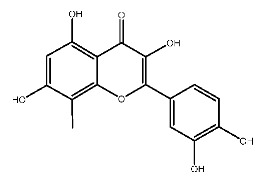	51.21	69.373	**11**	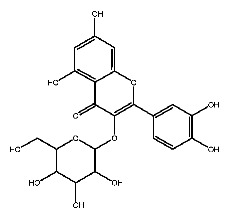	37.16	42.66
**3**	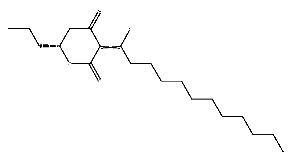	52.91	68.37	**12**	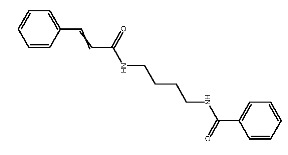	33.44	41.50
**4**	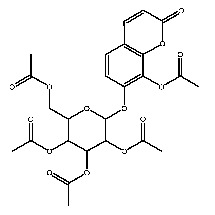	46.98	67.25	**13**	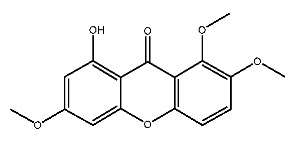	22.62	40.44
**5**	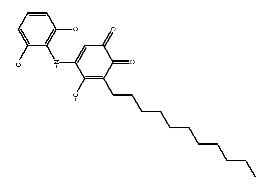	52.07	60.11	**14**	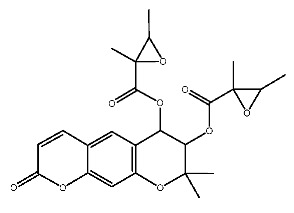	23.53	37.57
**6**	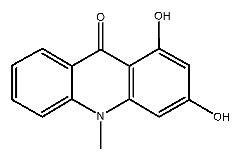	33.14	48.97	**15**	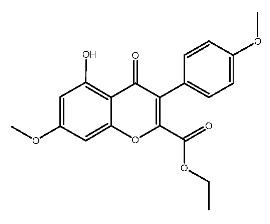	23.49	35.47
**7**	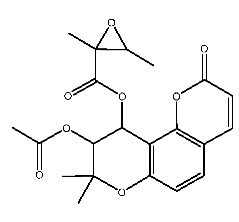	31.76	48.79	**16**	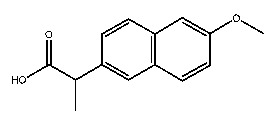	18.73	35.45
**8**	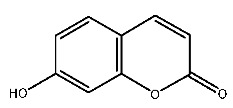	30.75	47.03	**17** ^a^	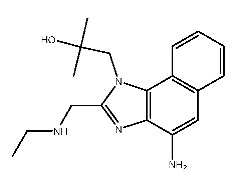	17.64	17.77
**9**	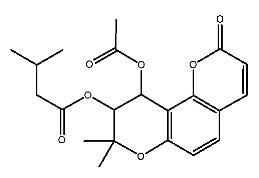	33.89	46.03	**18** ^b^	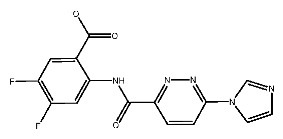	18.17	34.92

^a^ gardiquimod. ^b^ SR717.

**Table 2:** Physicochemical and drug-like properties of top 16 compounds.

No.	MW ^a^	HBA ^b^	HBD ^c^	RB ^d^	TPSA (Å^2^) ^e^	Lipinski #Violations	Bioavailability Score	Synthetic Accessibility
**1**	366.53	4	1	15	63.60	0	0.85	4.71
**2**	316.26	7	5	1	131.36	0	0.55	3.42
**3**	338.48	4	1	13	63.60	0	0.85	4.48
**4**	550.47	14	0	13	180.17	2	0.17	5.72
**5**	438.39	3	2	12	66.40	0	0.85	4.01
**6**	241.24	3	2	0	62.46	0	0.55	1.86
**7**	402.39	8	0	5	104.57	0	0.55	4.88
**8**	162.14	3	1	0	50.44	0	0.55	2.56
**9**	388.41	7	0	6	92.04	0	0.55	4.58
**10**	270.24	5	3	1	90.90	0	0.55	2.96
**11**	464.38	12	8	4	210.51	2	0.17	5.32
**12**	322.4	2	2	10	58.20	0	0.55	2.48
**13**	302.28	6	1	3	78.13	0	0.55	3.27
**14**	458.46	9	0	6	117.10	0	0.55	5.25
**15**	370.35	7	1	6	95.20	0	0.55	3.58
**16**	230.26	3	1	3	46.53	0	0.85	1.85

^a^ MW, molecular weight; ^b^ HBA: number of H-bond acceptors; ^c^ HBD: number of H-bond acceptors; ^d^ RB: number of rotatory bonds; ^e^ TPSA: topological polar surface area.

**Table 3:** MM-GBSA binding free energies (kcal/mol).

Complex	Δ*E*_vdW_	Δ*E*_ele_	Δ*G*_GB_	Δ*G*_SA_	Δ*G*_Bind_^a^
pTLR7-gardiquimod	−38.96 ± 1.17	−172.62 ± 6.41	190.75 ± 5.68	−4.37 ± 0.16	−25.21 ± 1.65
pSTING-SR717	−33.07 ± 1.48	−49.65 ± 21.88	59.86 ± 21.41	−5.21 ± 0.29	−27.97 ± 2.14
pTLR7-compound **2**	−33.28 ± 3.09	0.00 ± 0.00	8.85 ± 1.51	−4.23 ± 0.41	−28.65 ± 2.13
pTLR7-compound **7**	−43.00 ± 3.76	−12.56 ± 13.11	25.51 ± 14.46	−5.53 ± 0.52	−35.93 ± 3.75
pSTING-compound **2**	−36.53 ± 5.42	−8.73 ± 15.12	19.57 ± 16.95	−4.78 ± 0.78	−30.12 ± 4.04
pSTING-compound **7**	−43.78 ± 1.94	−27.18 ± 6.56	45.08 ± 4.94	−5.81 ± 0.17	−31.70 ± 0.88

^a^ The binding energy of complexes was calculated based on three replicas of MD simulations, and the values were expressed as mean ± standard deviation.

**Table 4:** The structures of derivatives and docking scores with two receptor proteins.

No.	Structure	−CDocker Energy (kcal/mol)		No.	Structure	−CDocker Energy (kcal/mol)	
		pTLR7	pSTING			pTLR7	pSTING
**2** **-** **1**	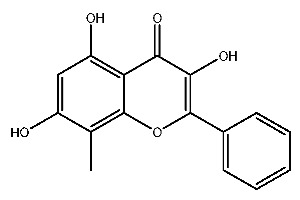	31.82	46.74	**7** **-** **1**	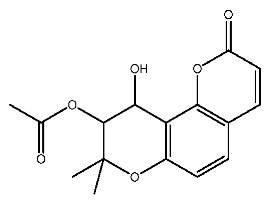	28.98	35.90
**2** **-** **2**	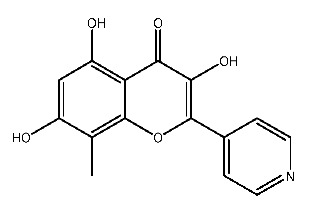	34.41	46.47	**7** **-** **2**	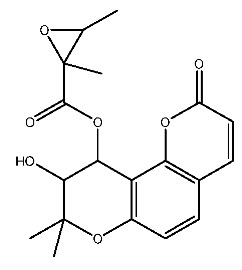	21.43	29.00
**2** **-** **3**	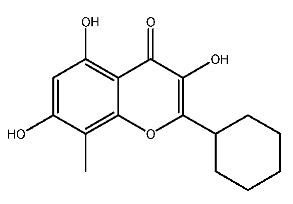	29.83	54.36	**7** **-** **3**	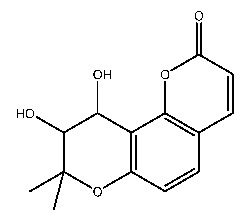	15.01	20.85
**2** **-** **4**	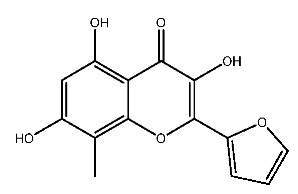	34.68	46.69	**7** **-** **4**	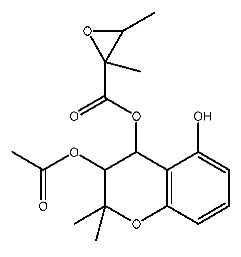	23.24	39.43
**2** **-** **5**	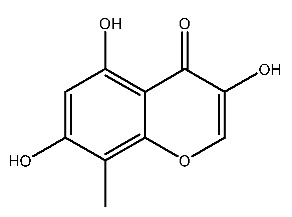	33.69	61.55	**7** **-** **5**	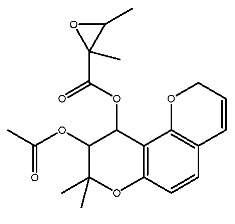	17.58	30.14
**2** **-** **6**	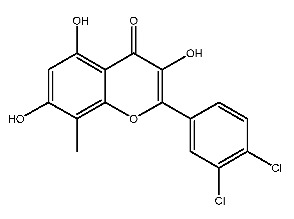	31.4588	43.3446	**7** **-** **6**	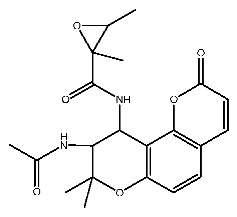	32.96	36.39
**2** **-** **7**	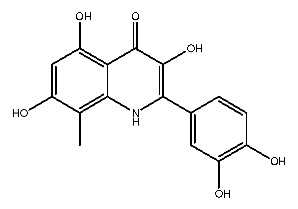	51.88	62.64	**7** **-** **7**	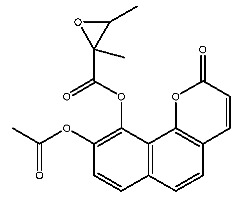	19.16	24.44
**2** **-** **8**	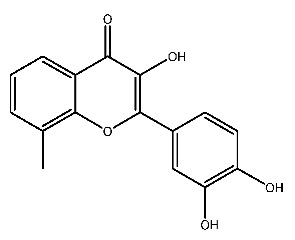	42.40	57.16	**7** **-** **8**	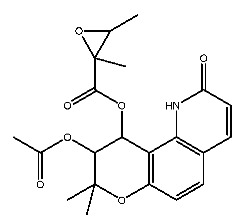	34.94	37.10
**2** **-** **9**	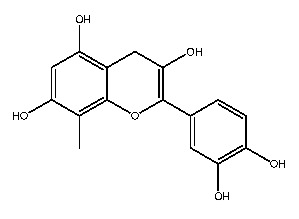	39.65	45.64	**7** **-** **9**	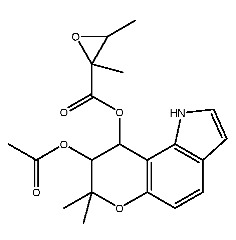	31.29	29.58
**2** **-** **10**	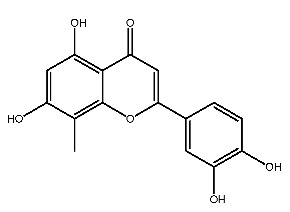	55.02	50.57				

## Data Availability

The original contributions presented in this study are included in the article. Further inquiries can be directed to the corresponding authors.

## Abbreviations

The following abbreviations are used in this manuscript:

TLR	Toll-like receptor 7
STING	Stimulator of Interferon Genes
MD	Molecular dynamics
CDNs	Cyclic dinucleotides
SMILES	Simplified Molecular Input Line Entry System
HBD	Hydrogen bond donors
HBA	Hydrogen bond acceptors
RB	Rotatable bonds
TPSA	Topological polar surface area
GI	Gastrointestinal
BBB	Blood–brain barrier
DTP	Developmental toxicity potential
LD_50_	Median lethal dose
LOAEL	Lowest observed adverse effect level
PME	Particle Mesh Ewald
NVT	Canonical ensembles
NPT	Isothermal isobaric ensembles
MM/GBSA	Molecular mechanics/generalized born surface area
RMSD	Root mean square deviation
RMSF	Root mean square fluctuation
Rg	Radius of gyration
SASA	Solvent accessible surface area
NHB	Hydrogen bond number

## Appendix A

**Table A1:** SMILES codes of several representative ligands of TLR7 and STING.

Ligands	SMILES
Guanosine	C1=NC2=C(N1[C@H]3[C@@H]([C@@H]([C@H](O3)CO)O)O)N=C(NC2=O)N
Imiquimod	CC(C)CN1C=NC2=C1C3=CC=CC=C3N=C2N
Resiquimod	CCOCC1=NC2=C(N1CC(C)(C)O)C3=CC=CC=C3N=C2N
Cpd-6	NC1=C2C(N(CC3=CC=C(CN4C[C@H]5N(C)C[C@@H]4C5)C=C3)C(C6=CN=CC(F)=C6)=N2)=NC(OCC)=N1
JRI	N#CC1=C(N=CC=C2)C2=C(N3C[C@H](C(N[C@@H]4[C@H](F)CNC4)=O)O[C@H](C)C3)C=C1
Purine analogs	CCN(CC1)CCN1CCCN2C3=NC=NC(N4CCN(CC)CC4)=C3N=C2
2′,3′-cGAMP	C1C2C(C(C(O2)N3C=NC4=C3N=C(NC4=O)N)OP(=O)(OCC5C(C(C(O5)N6C=NC7=C(N=CN=C76)N)O)OP(=O)(O1)O)O)O
di-ABZI	CCN1C(=CC(=N1)C)C(=O)NC2=NC3=C(N2CCN4C5=C(C=C(C=C5)C(=O)N)N=C4NC(=O)C6=CC(=NN6CC)C)C=CC(=C3)C(=O)N
SR-717	C1=CC(=NN=C1C(=O)NC2=CC(=C(C=C2C(=O)[O−])F)F)N3C=CN=C3
Astin-C	O=C(N[C@@H](CC)C1=O)C[C@@H](NC([C@@H](NC([C@@H](NC([C@H]2N1C[C@@H](Cl)[C@H]2Cl)=O)CC)=O)CO)=O)C3=CC=CC=C3
C-178	NC1=CC=C(C(NC2=CC=C(C(C=CC=C3)=C3O4)C4=C2)=O)O1
H-151	O=C(NC1=CC=C(CC)C=C1)NC2=CNC3C2C=CC=C3

**Table A2:** Docking score and RMSD value of re-docking.

PDB ID	−CDocker Energy (kcal/mol)	RMSD
5GMF	10.22	0.33
6YIF	91.99	0.36
7SHP	41.84	1.95
4KBY	87.37	0.01

**Table A3:** Solubility and pharmacokinetic properties of 16 compounds.

No.	Consensus Log Po/w	Log S (ESOL)	ESOL Class	GI ^a^ Absorption	BBB ^b^ Permeant	P-gp ^c^ Substrate	CYP1A2 Inhibitor	CYP2C19 Inhibitor	CYP2C9 Inhibitor	CYP2D6 Inhibitor	CYP3A4 Inhibitor
**1**	5.64	−6.19	Poorly soluble	High	No	No	Yes	Yes	Yes	No	No
**2**	1.66	−3.84	Soluble	High	No	No	Yes	No	No	Yes	Yes
**3**	4.91	−5.47	Moderately soluble	High	No	No	Yes	Yes	Yes	Yes	No
**4**	1.60	−3.37	Soluble	Low	No	No	No	No	No	No	Yes
**5**	6.13	−7.32	Poorly soluble	Low	No	No	No	Yes	Yes	No	Yes
**6**	2.05	−3.78	Soluble	High	Yes	No	Yes	No	No	Yes	Yes
**7**	2.57	−3.69	Soluble	High	No	No	No	No	No	No	Yes
**8**	1.51	−2.46	Soluble	High	Yes	No	Yes	No	No	No	No
**9**	3.23	−4.15	Moderately soluble	High	No	No	No	Yes	Yes	No	Yes
**10**	2.11	−3.94	Soluble	High	No	No	Yes	No	No	Yes	Yes
**11**	−0.25	−3.04	Soluble	Low	No	No	No	No	No	No	No
**12**	3.21	−3.63	Soluble	High	Yes	No	Yes	Yes	Yes	Yes	Yes
**13**	2.46	−3.92	Soluble	High	Yes	No	Yes	No	Yes	Yes	Yes
**14**	2.85	−4.14	Moderately soluble	High	No	No	No	No	No	No	Yes
**15**	3.16	−4.54	Moderately soluble	High	No	No	Yes	No	Yes	No	Yes
**16**	2.76	−3.61	Soluble	High	Yes	No	No	No	No	No	No

^a^ GI, gastrointestinal absorption; ^b^ BBB, blood–brain barrier; ^c^ P-gp, P-glycoprotein.

**Table A4:** Predicted toxicity of 16 compounds.

No.	Hepatotoxicity	Rat Oral LD_50_ (g/kg)	LD_0_ (g/kg)	Rat NTP ^a^		Ames Mutagenicity	LOAEL (g/kg)	DTP
				Female	Male			
**1**	FALSE	5.412	0.432	Non-Carcinogen	Non-Carcinogen	Non-Mutagen	0.045	Non-Toxic
**2**	TRUE	1.474	1.327	Non-Carcinogen	Carcinogen	Mutagen	0.218	Toxic
**3**	FALSE	4.413	0.378	Non-Carcinogen	Non-Carcinogen	Non-Mutagen	0.046	Non-Toxic
**4**	TRUE	1.440	0.024	Non-Carcinogen	Carcinogen	Non-Mutagen	0.028	Toxic
**5**	FALSE	2.582	0.807	Non-Carcinogen	Non-Carcinogen	Non-Mutagen	0.119	Non-Toxic
**6**	TRUE	1.122	0.293	Carcinogen	Non-Carcinogen	Mutagen	0.060	Non-Toxic
**7**	TRUE	0.245	0.024	Non-Carcinogen	Non-Carcinogen	Non-Mutagen	0.015	Toxic
**8**	TRUE	0.858	0.207	Non-Carcinogen	Carcinogen	Non-Mutagen	0.052	Toxic
**9**	FALSE	0.527	0.043	Non-Carcinogen	Non-Carcinogen	Non-Mutagen	0.010	Toxic
**10**	TRUE	0.748	0.541	Non-Carcinogen	Carcinogen	Non-Mutagen	0.065	Toxic
**11**	FALSE	0.846	1.756	Non-Carcinogen	Carcinogen	Non-Mutagen	0.075	Toxic
**12**	TRUE	6.690	0.209	Non-Carcinogen	Non-Carcinogen	Non-Mutagen	0.229	Toxic
**13**	TRUE	0.545	0.165	Non-Carcinogen	Carcinogen	Mutagen	0.072	Toxic
**14**	TRUE	0.365	0.019	Non-Carcinogen	Carcinogen	Non-Mutagen	0.007	Toxic
**15**	TRUE	0.500	0.133	Non-Carcinogen	Carcinogen	Non-Mutagen	0.037	Toxic
**16**	TRUE	0.556	0.208	Carcinogen	Non-Carcinogen	Non-Mutagen	0.051	Non-Toxic

^a^ NTP, U.S. national toxicology program.

**Table A5:** CoM distances of receptor–ligand complexes.

Complex	CoM Distance (Å)				
	0 ns	25 ns	50 ns	75 ns	100 ns
pTLR7-Gardiquimod	3.97	2.36	1.13	2.68	3.77
pTLR7-Compound 2	3.27	5.53	6.00	6.19	4.12
pTLR7-Compound 7	2.80	2.66	3.45	3.57	2.33
pSTING-SR717	1.82	1.54	2.76	2.11	3.39
pSTING-Compound 2	2.17	2.30	1.81	1.73	4.45
pSTING-Compound 7	2.56	1.94	1.68	1.27	3.04

**Table A6:** Sequence identity of TLR7 and STING between pigs, humans, and mice.

Protein	Accession	Species	Percent Identity	Query Cover	E-Value
TLR7	AAZ99026.1	Human	84.95%	100%	0
TLR7	AAI32386.1	Mouse	78.29%	100%	0
STING	NP_938023.1	Human	76.98%	100%	0
STING	NP_082537.1	Mouse	69.92%	100%	0
